# Antimicrobial Resistance, Enterotoxin and *mec* Gene Profiles of *Staphylococcus aureus* Associated with Beef-Based Protein Sources from KwaZulu-Natal Province, South Africa

**DOI:** 10.3390/microorganisms10061211

**Published:** 2022-06-14

**Authors:** Thembeka Thwala, Evelyn Madoroba, Tsolanku S. Maliehe, Kudakwashe Magwedere, Albert K. Basson, Patrick Butaye

**Affiliations:** 1 Department of Biochemistry and Microbiology, University of Zululand, Private Bag X1001, KwaDlangezwa, Empangeni 3886, South Africa; thembekaprincess6@gmail.com (T.T.); madorobae@unizulu.ac.za (E.M.); maliehet@unizulu.ac.za (T.S.M.); bassona@unizulu.ac.za (A.K.B.); 2 Directorate of Veterinary Public Health, Department of Agriculture, Land Reform and Rural Development, Pretoria 0001, South Africa; kudakwashem@dalrrd.gov.za; 3 Department of Pathobiology, Pharmacology and Zoological Medicine, Faculty of Veterinary Medicine, Ghent University, Salisburylaan 133, B9820 Merelbeke, Belgium; 4 Department of Biomedical Sciences, Ross University School of Veterinary Medicine, Basseterre P.O. Box 334, Saint Kitts and Nevis

**Keywords:** methicillin-resistant *Staphylococcus aureus*, staphylococcal enterotoxin genes, antimicrobial resistance, beef-based products, *S. aureus* contamination, food safety

## Abstract

Annually, approximately 23,000 cases of food poisoning by *Staphylococcus aureus* enterotoxins are reported worldwide. The aim of this study was to determine the occurrence and characterize *S. aureus* on beef and beef products in South Africa. Organ meats (*n* = 169), raw processed meat (*n* = 110), raw intact (*n* = 53), and ready-to-eat meats (*n* = 68) were obtained from 25 retail outlets. *S. aureus* was isolated and enumerated according to the ISO 6888-1 method. Identification of the strains was performed by MALDI-TOF MS. The antimicrobial resistance was determined using the disc diffusion test. The presence of methicillin-resistance genes and the staphylococcal enterotoxin genes was determined by PCR. Prevalence was low (13/400; CI 1.7–5) and all but one positive sample were from organ meats. Eight isolates were resistant to at least one antibiotic. Two isolates carried the *mecC* gene. All the isolates tested positive for *seg*, *seh*, *sei*, and *sep*, whilst 53.8% were positive for *sea*. None of the isolates was positive for *ser*, *sej*, *seb*, *sec*, or *sed*. The prevalence of *S. aureus* was low, with organ meats being the most contaminated. The presence of *mecC*-positive MRSA and of enterotoxins warrants further investigation and risk assessment.

## 1. Introduction

Beef is known for its role in supplying protein, minerals, and vitamins in human nutrition [1]. Due to its high nutritional content, beef is an excellent substrate for the growth of microorganisms, of which some are leading causes of meat spoilage [2]. Spoilage of meat is enhanced by inadequately stored or packed meat [3]. Different storage conditions, such as cold and gaseous composition, on packed meat are most likely to suppress the microflora, among them *S. aureus* [4].

*S. aureus* causes staphylococcal food poisoning (SFP) through the ingestion of food contaminated with staphylococcal enterotoxins [5]. This enterotoxaemia is characterized by diarrhea, nausea, abdominal cramping, and vomiting within 24 h of eating [6]. Contamination of food by *S. aureus* may originate from the animal, the food handlers, and the environment. It may be a consequence of poor hygiene during processing from slaughter to final product or inappropriate storage and household manipulations; however, contamination of meat is a complicated process which may occur well before the meat reaches retail outlets [7]. In addition to toxins encoded by the *seb*, *sec*, *sed*, and *see* genes, in particular, strains that produce the Staphylococcal Enterotoxin A (SEA), encoded by the *sea* gene, have caused a large number of outbreaks [8,9].

Antimicrobial resistance (AMR) is an increasing global challenge mainly driven by the overall use of antimicrobials [10]. In certain *S. aureus* clones, AMR is a major problem, especially in methicillin-resistant *S. aureus* (MRSA), of which the prevalence increases globally [11]. In the last 15 years, MRSA clonal complex 398 was discovered in food-producing animals, while other sequence types (ST), such as ST1, ST5, ST9, ST97, ST130, and ST433, have been reported to a lesser extent [12]. These strains were subsequently named Livestock Associated (LA)-MRSA. LA-MRSA Clonal complex 398 (CC 398) is mainly prevalent in Europe and North America; however, it has been reported in Asia as well as in Africa [12]. To a lesser extent, LA-MRSA CC398 has been associated with different infections in humans, including skin and soft tissue illnesses, ventilator-associated pneumonia, and septicemia [13].

There are few studies that have been conducted on meat and meat products in Africa so far [14,15]; most studies did not type the isolates, and only few of the studies have identified AMR genes [14,15,16,17]. Therefore, this study aimed to determine the occurrence, AMR, and virulence genes of *S. aureus* isolated from beef and beef products in retail outlets of the KwaZulu-Natal province, South Africa.

## 2. Materials and Methods

### 2.1. Ethical Approval

Ethical approval for this study was obtained from the University of Zululand with certificate number UZREC 171110-030 PMG 2019/112.

### 2.2. Study Design

**Figure 1:**
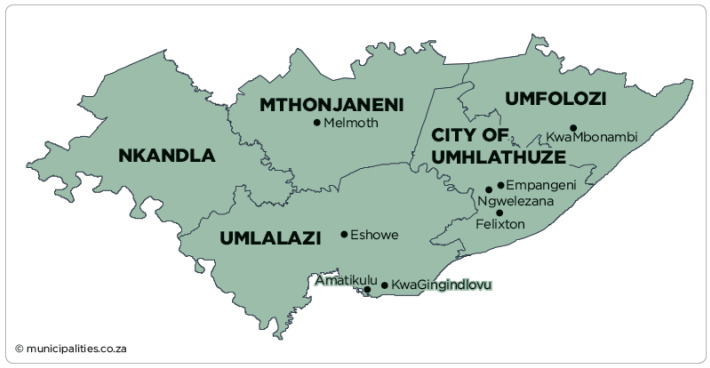
Geographical map representing King Cetshwayo district, KwaZulu-Natal. Source: https://municipalities.co.za/map/124/king-cetshwayo-district-municipality (accessed on 1 November 2020) [19].

**Figure 2:**
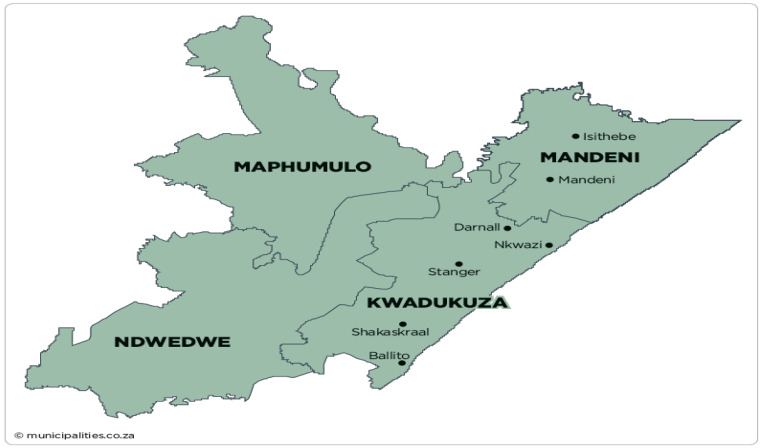
Geographical map representing iLembe district, KwaZulu-Natal. Source: https://municipalities.co.za/map/117/ilembe-district-municipality (accessed on 1 November 2020) [19].

The cross-sectional study involved the collection and microbiological analysis of meat and meat products from retail outlets and butcheries from King Cetshwayo and iLembe districts in the KwaZulu-Natal province of South Africa. King Cetshwayo district covers an area of 8213 km^2^ from the north coast region, whereas iLembe covers an area of 3269 km^2^ from the south coast region with a population of approximately 885,944 and 606,809, respectively [18]. The two districts contribute to about a quarter of the total population in KwaZulu-Natal KZN (Figure 1 and Figure 2).

### 2.3. Sample Size Determination

There are important statistical variables to consider when determining the sample size for a surveillance study [20]. These include z, α, p, and d, where z (1.96) is the normal deviate for two-tailed alternative hypotheses [21]. Alpha (α) is the level of significance and it is usually 5%, which implies that having a 5% probability of incorrectly rejecting a null hypothesis is acceptable [22]. The *p*-value is the expected prevalence proportion, and a prevalence of 50% (0.50) was assumed in this study based on a national surveillance of foodborne pathogens in South Africa by [23], which detected about 56% *S. aureus* in diverse meat and meat products from various establishments. The value of d is precision, and at the confidence interval of 95%, d is 0.05. In this study, the following formula was used to calculate the sample size of the surveillance study:

(1) Sample size =z(1−α2)2p(1− p)d2=1.962 0.50(1−0.50)0.052=384

However, 400 samples were collected in this study for robust results.

### 2.4. Sample Collection

A total of 400 samples were collected during the cross-sectional study. Twenty-five retail outlets and butcheries from King Cetshwayo and iLembe districts were included in this study. The beef samples were ready-to-eat beef products (*n* = 68), raw processed beef (*n* = 110), raw intact beef (*n* = 53), and organ meats (*n* = 169). Samples were packed into sampling bags using strict aseptic techniques, and placed in cooler bags containing ice packs to maintain a temperature of approximately 4 °C. The packaged samples were transported immediately to the Microbiology laboratory at the University of Zululand, Department of Biochemistry and Microbiology, for further bacteriological examination. Samples were analyzed immediately after arrival.

#### 2.4.1. Microbiological Analysis

##### Control Strains for Quality Control

The *S. aureus* ATCC 25,923 (Microbiologics, MN, USA) and field strains (positive for tested virulence factors) were included in all laboratory experiments as positive control strains. ATCC 25,922 was used as negative control.

##### Detection, Enumeration, and Isolation and Identification of *S. aureus*

The detection, enumeration, and isolation of *S. aureus* was performed according to the ISO 6888-1:1999 AMD 2018 standard method [24]. Briefly, each sample was analyzed for the presence of *S. aureus* by weighing 25 g, followed by addition of 225 mL of buffered peptone water. The samples and buffered peptone water were thoroughly mixed in a homogenizer (Bagmixer 400 cc, Interscience, France) for 2 min at 10 stroke/s. Subsequently, ten-fold serial dilutions were made using sterile pipettes [25]. From these dilutions, 0.1 mL was inoculated in duplicate onto Baird Parker agar plates (Oxoid, UK) using the spread plate technique, as described by Goja et al. [25]. Plates were incubated at 37 °C for 24 to 48 h. After incubation, the typical colonies were counted. Typical *Staphylococcus* spp. appeared as shiny black colonies [26]. To calculate the number of colony-forming units per gram (CFU/g), the colonies on the countable plate were multiplied by final dilution factor. The presumptive colonies were purified three times through sub-culturing on nutrient agar (Oxoid, UK) and incubation at 37 °C for 24 h. The presumptive *S. aureus* colonies were subjected to Gram staining, catalase test, mannitol salt, and free and bound coagulase tests (Oxoid, UK) [27]. Gram-positive cocci that appeared purple with grape-like shape were catalase-positive, appearing yellow on mannitol salt agar due to mannitol fermentation, and where coagulase-positive, were considered to be presumptive *S. aureus* and the colonies were subjected to further tests. Identification of *S. aureus* was confirmed using MALDI-TOF MS, according to the manufactures instructions for the MALDI Biotyper® (Bruker Daltonics, Germany) [28]. All confirmed *S. aureus* isolates were streaked on 5% sheep blood agar plates to identify the type of hemolysin they produce [29]. The plates were incubated at 37 °C for 24 h ± 2 [30].

### 2.5. Antimicrobial Susceptibility Testing

For the antimicrobial susceptibility test, the Kirby Bauer disk diffusion method according to Clinical Laboratory Standard Institute guidelines was applied [31,32]. Briefly, from a pure bacterial culture, 2–5 colonies were suspended in 5 mL sterile saline solution. The bacterial concentration was adjusted to an optical density of 0.5 on the McFarland scale [31,33]. A sterile cotton swab was dipped into the suspension and excess fluid was removed by squeezing the swab at the top of the bijou bottles. The bacteria were inoculated onto Mueller Hinton agar (Thermofisher, Waltham, MA, USA) by streaking in three different directions to obtain confluent bacterial growth. The medium surface was allowed to dry, followed by placing the following antimicrobial disks: ciprofloxacin (5 µg), cefoxitin (30 µg), clindamycin (2 µg), erythromycin (15 µg), rifampicin (5 µg), oxacillin (1 µg), kanamycin (30 µg), penicillin G (10 units), chloramphenicol (30 µg), gentamicin (10 µg), and trimethoprim (25 µg) (Davies Diagnostics, Randburg, South Africa) [34]. 

### 2.6. Detection of Selected Resistance and Virulence Genes

#### 2.6.1. DNA Extraction and PCR for Staphylococcal Enterotoxins and *mec* Genes

The Zymo DNA extraction kit (California, CA, USA) was used for the extraction of the DNA according to the manufacturer’s instructions. The quality and quantity of the DNA were measured using a Nanodrop 2000 spectrophotometer (Thermofisher Scientific, Waltham, MA, USA).

The enterotoxin genes (*sea*, *seb*, *sed*, *sec*, *she*, *seg*, *ser*, *sei*, *sep*, *sej*) and *mec* genes (*mecA* and *mecC*) were assessed by PCR using the primers listed in Table 1. The 20 µL PCR reaction mixtures contained 10–30 ng of template DNA (in 1 µL), NEB one Taq 2× master mix with standard buffer (10 µL), forward primer (1 µL), reverse primer (1 µL), and nuclease free water (7 µL). PCR amplifications for *sea*, *seb*, *sec*, *sed*, and *ser* were carried out in a thermal cycler with the following thermal conditions: initial denaturation for 5 min at 95 °C; 35 cycles of 30 s at 94 °C, 40 s at 56 °C, and 1 min at 68 °C; final extension for 5 min at 68 °C. The PCR conditions for *seg*, *sei*, *sep*, *sej* and *she* were similar to the above, except that the annealing stage was performed at 53 °C for 40 s.

#### 2.6.2. Agarose Gel Electrophoresis

PCR products were subjected to electrophoresis in 1.5% agarose gels stained with ethidium bromide at 3 volts/cm for approximately 30 min. The 50 bp and 100 bp DNA ladders were used to estimate the size of PCR amplicons. The PCR amplicons were visualized under ultraviolet light and the gel images were documented using a gel documentation system (E-Box).

## 3. Results

### 3.1. Prevalence of S. Aureus in Meat

Out of the 400 beef and beef products that were analyzed, 3.25% (*n* = 13; CI 1.7–5) tested positive for *S. aureus* (Table 2). From each of the 13 positive samples, one isolate was retained for further investigation. The *S. aureus*-positive samples were predominantly organ meats (*n* = 10/13; CI 46.2–95), followed by raw intact beef (*n* = 2/13; 1.9–45). Only one of the 13 *S. aureus*-positive samples was from ready-to-eat beef. No *S. aureus* was detected in raw processed beef. The 13 *S. aureus* from 13 positive samples showed alpha, beta, and gamma hemolysis reactions on 5% sheep blood agar.

### 3.2. Enumeration of Staphylococcus aureus

Table 3 shows the results of *Staphylococcus aureus* enumeration of the 13 positive samples. The *S. aureus* counts from beef-based products ranged from 2.65 log_10_ CFU/g to 4.1 log_10_ CFU/g (Table 4). *S. aureus* from 1 of the 13 positive samples (ox kidneys) were too numerous to count.

### 3.3. Antimicrobial Susceptibility Testing

AMR of the *S. aureus* isolates are shown in Table 4. Eight out of 13 (61.54%) isolates were resistant to at least one antibiotic. Less than 50% of the isolates exhibited resistance to penicillin G (38.46%; *n* = 5/13; CI 13.9–68), cefoxitin (7.69%; *n* = 1/13; CI 0.2–36), tetracycline (7.69%; *n* = 1/13; 0.2–36), oxacillin (7.69%; *n* = 1/13; CI 0.2–36), clindamycin (30.76%; *n* = 4/13; CI 9.1–61), erythromycin (23.07%; *n* = 3/13; CI 5–54), ciprofloxacin (15.38%; *n* = 2/13; CI 1.9–45), and rifampicin with a resistance percentage of 7.6%. (*n* = 1/13; CI 0.2–36). Multi-drug resistance (MDR), which is the lack of susceptibility to at least three antimicrobial classes [40], was observed in two *S. aureus* isolates. Two MDR profiles were observed, namely, PG-FOX-OX-RP-CD-E-TET (*n* = 1) and PG-E-CD (*n* = 1).

### 3.4. Detection of Selected Resistance and Virulence Genes

#### 3.4.1. Methicillin-Resistant Determinants

**Figure 3:**
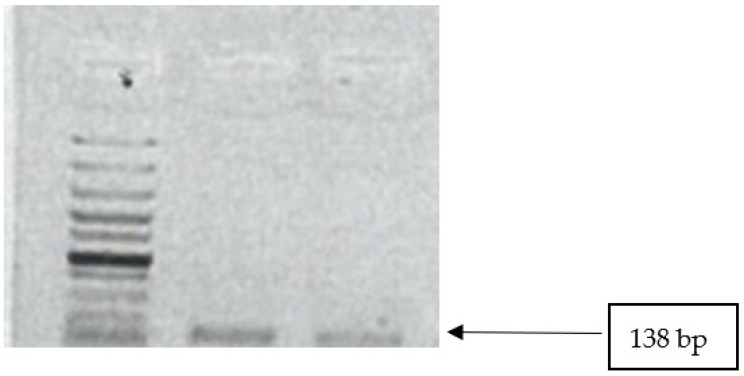
Image showing *mecC* gene amplicons observed on agarose gel. Lane 1: 100 bp DNA ladder; lanes 2–3 show positive band for *mecC* genes (138 bp).

All isolates were tested for the presence of *mecA* and *mecC* genes. None of the isolates were positive for *mecA* genes. Two isolates (15.4%; CI 1.9–45) tested positive for *mecC* gene (Figure 3).

#### 3.4.2. *S. aureus* Enterotoxin Genes

**Figure 4:**
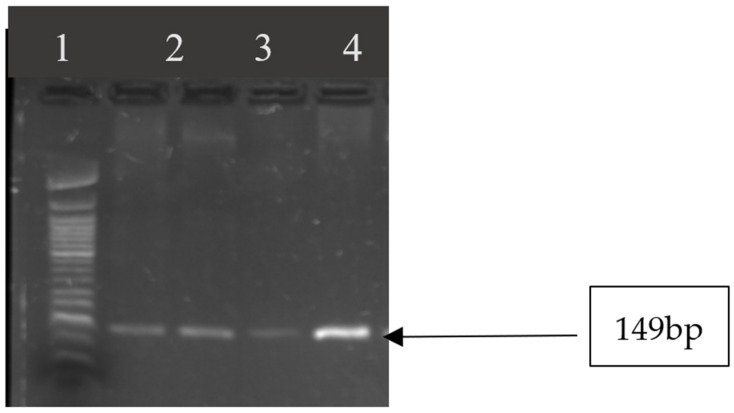
Image showing *seg* gene amplicons observed on agarose gel. Lane 1: 50 bp DNA ladder; lanes 2–5 show amplicon sizes for samples that were positive for *seg* gene (149 bp).

**Figure 5:**
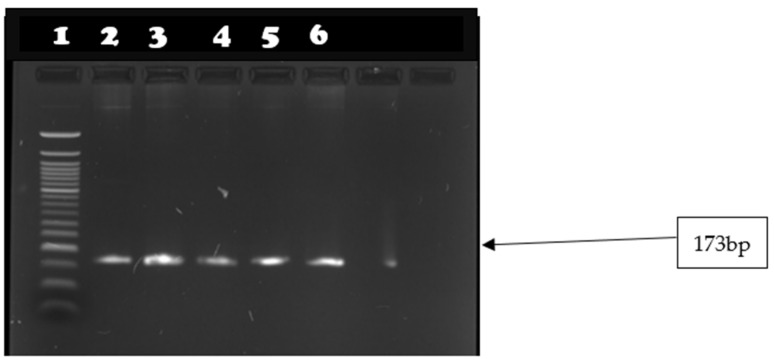
Image showing *seh* gene amplicons observed on agarose gel. Lane 1: 50 bp DNA ladder; lanes 2–6 show amplicon sizes for samples that were positive for *seh* gene (165 bp).

In Table 5, Figure 4 and Figure 5, the results of the virulence genes are shown. Out of eleven enterotoxin genes that were tested, five (*sep*, *seh*, *sei*, *sej*, *sea*) were detected. All *S. aureus* strains were positive for *seg*, *seh*, and *sei*, *sep*. The *sea* gene was detected in 7 of the 13 *S. aureus* (53.84%).

## 4. Discussion

The aim of this study was to determine the occurrence, AMR, and virulence characteristics of *S. aureus* from products of bovine origin in retail outlets of selected municipalities. Though most studies in Africa found prevalences of between 15 and 40%, with the exception of 55% *S. aureus* detection in Algeria, the overall occurrence of 3.25% from this study is lower compared to most of the previous African studies [41,42,43,44,45]. Higher prevalences of up to 65% have also been found in non-African countries such as Turkey, Jordan, United States of America (USA), and several countries in Europe [46,47,48,49,50,51,52]. It is important to note that differences in methodology and sample size should be taken into account and may explain, in part, the differences seen [53,54]. It is important to apply a system such as the ISO standard, used in this study, to allow direct comparison of the prevalence between different studies.

The relatively high *S. aureus* prevalence in organ meats (kidneys, livers, lungs) compared to raw intact beef meat and ready to-eat meat was conspicuous in this study (though not to a significant extent). Probably the organ meat may be more prone to cross contamination compared to other meat types, and *S. aureus* can be found in the intestines [55]. *S. aureus* occurrence was also observed in ready to-eat meats and this may be attributed to cross contamination and growth due to further preparation [56,57]. The differences in preparation and the type of preparation of the ready-to-eat (RTE) beef, as well as conservation of the product, may play a large influence.

The average counts of the *S. aureus*-positive samples in this study ranged from 2.65 log_10_ and 4.07 log_10_ per gram in organ meat. These *S. aureus* counts are lower than those that were previously observed for organ beef meat in South Africa (5.1 log–log 5.6) [14]. When considering the *S. aureus* limit of 100 CFU/g in RTE, proposed by the guidelines for environmental health officers on the interpretation of microbiological analysis data of food [58], the 13 positive samples were not within the compliance limits, though they were sold at retail level. The situation is concerning for RTE biltong, which is not processed further prior to consumption. The contaminated samples were mainly plucked meats that might not be subjected to similar strict hygiene scrutiny as beef cuts. It is possible that the *S. aureus* counts may have increased in the pluck meats during transportation, probably due to an inadequate cold chain, or the level of preservation at the retail level may have contributed to an increase in bacterial numbers.

As we found only few strains, comparing with other studies is difficult. The isolates from this study were, in general, more susceptible to antimicrobials than those from other studies on beef in South Africa [15] and other African countries [42,44], but are similar to studies in Europe [50], and higher than what has been detected in the United States of America [59].

Interestingly, two isolates were MRSA, with only one detected phenotypically. Phenotypic methicillin resistance should always be confirmed by PCR, as false positive and false negative results may be obtained by the phenotypic tests. While this may not seem a lot, it may have a significant public health impact. The MRSA isolates from this study were *mecC*-positive. While this resistance gene has not been associated extensively with MRSA either in humans or animals, it has, however, been isolated mainly from animals, including wildlife [60]. In most countries, the *mecA* gene is mostly found in MRSA [15,61,62,63,64,65]. However, in South Africa, the *mecC* gene has also been shown as the sole methicillin-resistance gene in strains from different food-producing animal species as well as wild birds [17]. This might indicate a very specific and unique situation in South Africa and urges for a more large-scale study of MRSA on food-producing animals, wild animals, and foods derived from animals in South Africa so as to determine the human health hazard. These studies should include whole-genome sequencing to determine their true epidemiology.

Staphylococcal enterotoxins (SEs) types SEA to SEE have been reported to account for approximately 95% of food poisoning outbreaks caused by staphylococci [66], whilst the remainder may be due to the other SE types, including SEG, SEH, SEI, SEJ, SEK, SEL, SEM, SEN, and SEO [5]. Based on the positive enterotoxin genes, it is clear that many *S. aureus* isolates from this study are enterotoxigenic. Some of the genes found in this study, such as *sea*, *seh*, *seg*, and *sei*, have been associated with outbreaks of food poisoning in different parts of the world [5,67,68,69,70]. However, *seg* and *sei* have not frequently been isolated from food isolates and are, rather, associated with staphylococcal scarlet fever and toxic shock syndrome [71]. The *seg*, *sei*, and *seh* have also been identified in patients with other *S. aureus*-associated infections [72].

In the current study, all the 13 *S. aureus* isolates tested positive for *seg*, *sei*, and *seh* genes. The *seg* and *sei* genes are components of the *egc* operon, together with *sem*, *sen*, and *seo* enterotoxin genes [70]. The *egc* operon is located on a mobile genetic element (MGE) [70] and can thus be transferred to non-pathogenic *S. aureus* [60]. This combination is, however, rarely found in strains involved in toxi-infections [5,38,46,71].

## 5. Conclusions

In conclusion, the current study contributes to the knowledge about *S. aureus* on beef in South African markets. While the overall prevalence was relatively low, care should, however, be taken when handling pluck meats to avoid cross contamination with utensils, working surfaces, and RTE. Few *S. aureus* isolates exhibited antimicrobial resistance; however, the presence of *mecC*-positive *S*. *aureus* strains is worrisome. Five classical staphylococcal enterotoxin genes were identified from these isolates, which indicate a health risk to the consumers. The observation of *mecC*-positive MRSA that are present on food and have been reported also in food-producing animals warrants a One Health study on MRSA in food-producing animals, pet animals, wildlife, and foods in South Africa. These studies should include whole-genome sequencing so as to determine the epidemiology and origins of *mecC*-positive MRSA.

## Figures and Tables

**Table 1:** Oligonucleotide sequence primers used to target genes for species confirmation in *S. aureus*.

Target Gene	Primer Sequence (5′-3′)	Product Size (bp)	Reference
*tsst*	5′ CATCTACAAACGATAATATAAAGG 3′ CATTGTTATTTTCCAA TAACCACCC	481	[35]
*mecA*	5′ GAA ATG ACT GAA CGT CCG AT 3′ CTG GAA CTT GTT GAG CAG AG	399	[35]
*sea*	5′ GGTTATCAATGTGCGGGTGG 3′ CGGCACTTTTTTCTCTTCGG	102	[36]
*seb*	5′ GTATGGTGGTGTAACTGAGC 3′ CCAAATAGTGACGAGTTAGG	164	[36]
*sec*	5′ AGATGAAGTAGTTGATGTGTATGG 3′ CACACTTTTAGAATCAACCG	451	[36]
*sed*	5′ CCAATAATAGGAGAAAATAAAA 3′ ATTGGTATTTTTTTTCGTTC	278	[36]
*ser*	5′ AGATGTGTTTGGAATACCCTAT 3′ CTATCAGCTGTGGAGTGCAT	123	[37]
*seg*	5′ GTTAGAGGAGGTTTTATG 3′ TTCCTTCAACAGGTGGAGA	198	[37]
*she*	5′ CAACTGCTGATTTAGCTCAG 3′ CCCAAACATTAGCACCA	173	[38]
*sei*	5′ GGCCACTTTATCAGGACA 3′ AACTTACAGGCAGTCCA	328	[37]
*sej*	5′ GTTCTGGTGGTAAACCA 3′ GCGGAACAACAGTTCTGA	131	[37]
*sep*	5′ TCAAAAGACACCGCCAA 3′ ATTGTCCTTGAGCACCA	396	[39]
*mecC*	5′ GAAAAAAAGGCTTAGAACGCCTC 3′ GAAGATCTTTTCCGTTTTCAGC	138	[17]

**Table 2:** Prevalence of *S. aureus* from beef-based products in selected districts from KZN.

Meat Type	Number of Samples	Number Positive	Prevalence (%)	CI *
Organ meat	169	10	5.9	2.9–11
Raw intact meat	53	2	3.8	0.5–13
Processed meat	110	0	0	0–3
Ready to-eat meat	68	1	1.5	0–8
Total	400	13	3.25	1.7–5

CI * refers to confidence intervals.

**Table 3:** *S. aureus* counts from beef and beef products.

Sample Number	Sample ID	Sample Type	Log_10_ CFU/g
176	KkwaAO176	Ox kidneys	3.82
167	KGinEO167	Ox tripe (bible)	4.07
174	KkwaAO174	Ox liver	3.65
200	KkwaLO200	Ox lungs	3.34
177	KkwaAO177	Ox kidneys	3.87
201	KkwaLO201	Ox lungs	3.62
235	KmelEI235	Beef steak tender	4.03
250	KmelDO250	Ox lungs	2.65
370	ILestanBR370	Biltong	3.37
98	KRbyEI98	Stewing beef	4.1
162	KGinEO162	Ox liver	3.38
238	KmelEO238	Ox liver	3.23
302	ILemanEO302	Ox kidneys	uncountable

**Table 4:** Antimicrobial resistance among 13 *S.* *aureus* (including MRSA) from meat and meat products in selected KZN province municipalities.

Antimicrobial Classes	Antimicrobial Agents	Number of Tested Isolates	Number of Resistant Isolates	Percentage % (CI)
Penicillins	Penicillin G	13	5	38.46 (13.9–68)
Penicillin-resistant penicillins	Oxacillin	13	1	7.69 (0.2–36)
Cephalosporins	Cefoxitin	13	1	7.69 (0.2–36)
Aminoglycosides	Gentamicin	13	0	0 (0–25)
	Kanamicin	13	0	0 (0–25)
Macrolides	Erythromicin	13	3	23.08 (5–54)
Lincosamides	Clindamicin	13	4	30.77 (9.1–61)
Fluoroquinolones	Ciprofloxacin	13	2	15.38 (1.9–45)
Phenicols	Chloramphenicol	13	0	0 (0–25)
Folate-pathway inhibitor	Trimethoprim	13	0	0 (0–25)
Rifampin	Rifampicin	13	1	7.69 (0.2–36)
Tetracyclines	Tetracycline	13	1	7.69 (0.2–36)

**Table 5:** PCR amplification results for *S. aureus* methicillin-resistance and enterotoxin genes.

Sample Code	Sample	*S. aureus* Enterotoxin, *mec* Genes and Resistance Profile														
		*sec*	*sed*	*seg*	*sep*	*sej*	*seh*	*sei*	*ser*	*sea*	*tsst*	*seb*	*sed*	*mecC*	*mecA*	R-Profile
KRbyEI98	Stewing beef	-	-	+	+	-	+	+	-	+	-	-	-	-	-	N
KGinEO162	Liver	-	-	+	+	-	+	+	-	+	-	-	-	+	-	N
KGinEO167	Tripe (omasum)	-	-	+	+	-	+	+	-	+	-	-	-	-	-	PG-CD-E
KkwaAO174	Liver	-	-	+	+	-	+	+	-	+	-	-	-	-	-	CIP
KkwaAO176	Kidneys	-	-	+	+	-	+	+	-	+	-	-	-	+	-	PG-FOX-TET-OX-CD-E-RP
KkwaAO177	Kidneys	-	-	+	+	-	+	+	-	+	-	-	-	-	-	PG-E
KkwaLO200	Lungs	-	-	+	+	-	+	+	-	-	-	-	-	-	-	N
KkwaLO201	Lungs	-	-	+	+	-	+	+	-	-	-	-	-	-	-	N
KmelEI235	Beef steak	-	-	+	+	-	+	+	-	-	-	-	-	-	-	CD
KmelEO238	Liver	-	-	+	+	-	+	+	-	-	-	-	-	-	-	PG
KmelDO250	Lungs	-	-	+	+	-	+	+	-	-	-	-	-	-	-	CD
ILemanEO302	Kidneys	-	-	+	+	-	+	+	-	-	-	-	-	-	-	PG-CIP
ILestanBR370	Biltong	-	-	+	+	-	+	+	-	+	-	-	-	-	-	N

N: susceptible to all antibiotics; R-profile: antimicrobial resistance profile for each isolate; FOX (cefoxitin); OX (oxacillin); PG (penicillin G); CIP (ciprofloxacin); TET (tetracycline); E (erythromycin); RP (rifampicin); CD (clindamycin).

## Data Availability

Not applicable.
